# Celiac Disease and Possible Dietary Interventions: From Enzymes and Probiotics to Postbiotics and Viruses

**DOI:** 10.3390/ijms231911748

**Published:** 2022-10-04

**Authors:** Sandip K. Wagh, Karen M. Lammers, Manohar V. Padul, Alfonso Rodriguez-Herrera, Veronica I. Dodero

**Affiliations:** 1 Department of Organic and Bioorganic Chemistry, Bielefeld University, 33615 Bielefeld, Germany; 2 Department of Biochemistry, Dr. Babasaheb Ambedkar Marathwada University, Aurangabad 431004, India; 3 Tubascan Ltd., 1098 XG Amsterdam, The Netherlands; 4 Department of Biochemistry, The Institute of Science, Dr. Homi Bhabha State University, Mumbai 400032, India; 5 Pediatrics, Saint Luke’s Hospital, University College Dublin, R95 FY71 Kilkenny, Ireland

**Keywords:** celiac disease (CeD), gut microbiota, dietary therapies, probiotics, glutenase, prebiotics, postbiotics, gluten, viromebiotics

## Abstract

Celiac Disease (CeD) is a chronic small intestinal immune-mediated enteropathy caused by the ingestion of dietary gluten proteins in genetically susceptible individuals. CeD is one of the most common autoimmune diseases, affecting around 1.4% of the population globally. To date, the only acceptable treatment for CeD is strict, lifelong adherence to a gluten-free diet (GFD). However, in some cases, GFD does not alter gluten-induced symptoms. In addition, strict adherence to a GFD reduces patients’ quality of life and is often a socio-economic burden. This narrative review offers an interdisciplinary overview of CeD pathomechanism and the limitations of GFD, focusing on current research on possible dietary interventions. It concentrates on the recent research on the degradation of gluten through enzymes, the modulation of the microbiome, and the different types of “biotics” strategies, from probiotics to the less explored “viromebiotics” as possible beneficial complementary interventions for CeD management. The final aim is to set the context for future research that may consider the role of gluten proteins and the microbiome in nutritional and non-pharmacological interventions for CeD beyond the sole use of the GFD.

## 1. Introduction

### 1.1. Celiac Disease: Gluten Proteins and the Triggers of Disease

Celiac Disease (CeD) is a chronic inflammatory, autoimmune-mediated disease affecting the duodenum [1]. It is accepted that gluten proteins found in wheat, rye, and barley are responsible for this autoimmune enteropathy. Celiac disease is remarkably ubiquitous, with epidemiologic data now available for every continent; unfortunately, data about Asia and Africa are limited, but the condition is present. Globally, the pooled seroprevalence of celiac disease is 1.4% (95% CI 1.1–1.7%). The prevalence of biopsy-confirmed celiac disease is 0.7% (95% CI 0.5–0.9%) [2,3]. CeD could be developed at any age where women are affected more frequently than men, with a ratio of 3:1 [4,5]. The typical clinical symptoms of CeD are chronic diarrhea, anemia, osteoporosis, and intestinal cramps. If left untreated, the disease may result in complications such as intestinal lymphoma and a slightly increased risk of early death [6,7,8]. Furthermore, the coexistence of CeD with mental disorders, such as schizophrenia [9] and type 1 diabetes mellitus, has also been reported [10,11]. If unaddressed, CeD can lead to villous atrophy and malabsorption of nutrients and, consequently, can cause symptoms such as anemia and osteoporosis [12].

CeD has emerged as a model disease to decipher how the interplay between environmental and genetic factors can predispose one to autoimmunity. It is still a challenge to integrate a full explanation of the considerable variability in disease penetrance, severity, and presentation of CeD. Many authors postulate gaps in the current knowledge and missing actors in the chain of pathophysiological events. An excellent comprehensive review of CeD immunopathogenesis has been recently published by Levescot et al. [13].

The keystone event in CeD pathogenesis is the activation of a gluten-specific immune response that is driven by molecular interactions between gluten-derived peptides, as the indispensable environmental factor, the HLA-DQ2/8 locus, as the main predisposing genetic factor, and the enzyme transglutaminase 2 (tTG2), the identified CeD-specific autoantigen.

The human gastrointestinal tract lacks luminal endo-prolylpeptidases, so the proline-rich gluten proteins are incompletely digested and can progress through the intestinal lumen. As a consequence, large immunogenic peptides can interact with the enzyme transglutaminase 2, which converts neutral and polar, glutamine residues into negatively charged glutamic acids. This transformation allows the positively charged amino acids in the HLA-DQ2 or HLA-DQ8 molecules at the surface of the antigen-presenting cells to bind with higher affinity to the gluten peptides harboring negative charges. These interactions trigger an expansion of gluten-specific CD4+ T cells that produce pro-inflammatory cytokines such as interleukins and interferon-gamma (IFN γ). The epithelial damage in the intestinal mucosa requires the activation of CD8+ cytotoxic intraepithelial lymphocytes (IEL).

Gluten proteins contain proline (P) and glutamine (Q) rich proteins, and due to repetitive PQ sequences, they are difficult to degrade for mammalian digestive tract enzymes [14,15]. The wheat protein gliadin (α/β-, γ- and ω-gliadins) is the primary toxic component of gluten [16] and the most studied protein related to CeD. Like gluten, gliadin is only partially degraded by human digestive enzymes [17,18]. Gliadins form different protein aggregates under the physiologically relevant pH of 3.0 and 7.0, enabling the enzymes’ accessibility to other possible degradation positions [19,20,21]. Recently, it was demonstrated that the gliadin peptides obtained after pepsin proteolysis behave as an amyloid-like structure associated with inflammatory cytokine production and pro-apoptotic mRNA expression [22]. The gliadin peptides that resist the human digestive and intestinal enzymes interact with enterocytes, opening the tight junctions that facilitate their transport to the lamina propria [23]. The pathogenic gliadin peptides are classified as “toxic” or “immunogenic,” depending on their behavior. If they induce intestinal damage in vivo and/or in vitro, they are designed as toxic peptides, and if they activate T cells, they are considered immunogenic peptides [24,25,26]. It is hypothesized that gliadin peptides in the lamina propria interact with tTG2 [27,28], the enzyme responsible for converting glutamine residues to glutamic acid. These deamidated peptides represent antigens interacting with increased affinity with the HLA (human leukocyte antigen)-DQ2 or HLA-DQ8 molecules. These molecules are part of the MHC (major histocompatibility complex) class II. The MHC II is an antigen-presenting receptor system by which antigen-presenting cells, like dendritic cells, present antigens (for instance, deamidated gliadin peptides) to T cells to elicit an immune response and build a memory of that specific antigen. The most studied immunodominant gliadin fragment is a 33 amino acids fragments, LQLQPFPQPQLPYPQPQLPYPQ-PQLPYPQPQPF, named 33-mer [29].

The amount of 33-mer that remains after proteolysis in different wheat cultivars was quantified as 91 to 603μg g−1 in wheat flour [30]. The 33-mer sequence is responsible for the adaptive immune response in CeD because it contains six partially overlapping copies of canonical T-cell epitopes [31], three copies of the DQ2.5-glia-α1- (PF/YPQPQLPY), and the DQ2.5-glia-α2 (PQPQLPYPQ) epitope [32]. It is accepted that the 33-mer reaches the lamina propria, where dendritic cells process and present it in the context of HLA-DQ2/8 to CD4+ T lymphocytes [33,34]. In recent years, it has been shown that the 33-mer forms dynamic small oligomers and large aggregates depending only on peptide concentration with a concomitant conformational transition to the parallel beta structure, which is the signature of amyloid proteins [35,36]. In vitro, the large 33-mer oligomers can trigger a Toll-like (TLR-2 and -4) receptor-mediated innate immune response in macrophages, showing the relevance of such structures in activating inflammation [37].

On the other hand, a sequestering polymer and non-absorbable (polyhydroxy ethyl methacrylate-co-styrene sodium sulfonate (P(HEMA-co-SS)) have been used to form larger complex gliadin particles to prevent the enzymatic action from stopping CeD activation [38]. It was proposed that the polymer-sequestered gliadin peptides would be discharged from the body, inhibiting their absorption and transport to the bloodstream [39,40]. This last research highlights the role of inhibiting the entrance of gluten peptides to the host as a therapeutic strategy.

A strong focus on the microbiota has been made, considering it first as an element involved in the CeD pathogenesis and, therefore, recently considered a potential intervention. Among the proposed mechanisms is the potential role of a positive feedback loop that may amplify the activation of nearby lymphocytes (IEL) induced by a type I interferon response triggered by viruses. Another hypothetical scenario is that a pathogen or a commensal bacterium might trigger a T cell cross-reaction with gluten peptide epitopes and drive an expansion of a cross-reactive T cell repertoire with epitope spreading [41]. An excellent example was recently reported where many mimics of HLA-DQ2.5-restricted gliadin determinants were found in the commensal bacterium *Pseudomonas fluorescens* that activate reactive T cells isolated from CeD patients. This report is major proof of the concept that a molecular mimicry mechanism may trigger CeD. Recently, high sequence similarity was found between 33-mer gliadin peptide and pathogen-derived proteins, e.g., extracellular proteins from *Streptococcus pneumoniae* and *Granulicatella* sp., by stringent BLASTp search [42]. Since *Granulicatella* sp. is found in the gut and has been reported in CeD patients, it would be interesting to investigate the role of this pathogen in the development of CeD by molecular mimicry mechanisms. Beyond T-cell activation, it has been hypothesized that gluten proteins have functional similarities with non-replicative pathogens such as prions [43]. It was also hypothesized that since gluten peptides share structural/morphological similarities with pathogens, they possess latent pathogenicity. Therefore, although initially innocuous to the host, their accumulation and oligomerization with the conformational transition toward amyloid-type structures could trigger their recognition by the host’s innate immunity [42].

Several viruses have been involved in CeD pathogenesis: the adenovirus 12 E1A, Enterovirus, Hepatitis C virus, and Rotavirus. However, their role was later dismissed, it has been described, for example, that the risk of CeD was increased in a cohort of children who combined being Enterovirus positive with a high gluten intake, indicating a cumulative effect of these two factors in the development of disease in genetically at-risk children [44]. Recently, Reovirus, which causes mostly asymptomatic infections, is the last virus to be suggested to have a role in CeD pathogenesis. Reovirus would break the tolerance to protein antigens [45]. The underlying pathogenetic mechanisms of early-life infections and CeD are not described but could provide new insights into the prevention of celiac disease.

### 1.2. Is a Gluten-Free Diet Enough for CeD Treatment?

Currently, the only treatment available for CeD is the strict elimination of gluten from the diet. This so-called gluten-free diet (GFD) usually results in the alleviation of symptoms and the improvement of small-bowel mucosal damage [46]. Several antibodies based on ELISAs are available to check for gluten traces in food products. R5 and G12 antibody-based ELISAs are frequently used to detect the threshold quantity of gluten (>20 mg/kg). The R5 monoclonal antibody (mAb) strongly recognizes the most toxic fragments of gliadin as QQPFP, QQQFP, LQPFP, and QLPFP sequences [47]. The G12 is a highly sensitive mAb antibody against the α2-gliadin 33-mer toxic peptide of the gliadin [48]. It has been proven to be efficient in measuring prolamin concentrations in native and partially hydrolyzed cereals. When both were compared, they showed no significant difference in the mean gluten concentration detected [6]. Each one offers different limitations depending on the food matrix analyzed.

Gluten exposure damages the intestinal epithelium, causing atrophy of the villous, which subsequently induces poor digestion and absorption of a range of macro and micronutrients. The classic picture of CeD was of skinny children with diarrhea. The current picture includes patients who are obese or show persistent constipation. Silvester et al. have compiled some myths and fallacies about the GFD. We know now that not all patients show the same level of response to a GFD. The poor response may happen because of hidden gluten exposure or coexisting conditions such as small intestinal bacterial overgrowth (SIBO) or autoimmune enteropathy. Other factors associated with symptomatic persistent villous atrophy include age > 70 years old and the use of proton-pump inhibitors (PPI), non-steroidal anti-inflammatory drugs, or selective serotonin reuptake inhibitors. Moreover, mucosal recovery on a GFD is not universal among those responding clinically. It was published that only one-third of adults have normal villi (healed and healthy intestine) after two years on a GFD. Only 2/3 will get it after five years on a GFD [49]. Of note, the data of some studies point to a constitutive defect of the intestinal barrier function. Diosdado et al. found an increased expression of innate and adaptive immune genes and markers of increased neutrophil activity in biopsies from CeD patients (active and in-remission) versus non-celiac individuals. Neutrophil recruitment was also visualized by immunohistochemistry. Although they observed a gradual change in immune gene expression after implementing a GFD until complete normalization, increased neutrophil recruitment was observed in both active and remission CeD patients. This result led the authors to conclude that despite clinical, histologic, and serologic normalization in remission, there was an ongoing activated innate immunity, and a link was proposed between the ongoing neutrophil recruitment and impaired barrier function [50]. Recently, [51] in cultured organoids from CeD patients, it wasfound various molecular markers of increased permeability, particularly decreased claudin-18, ZO-1, mucin components, trefoil factors, and an increased expression of claudin-2. In addition, they determined that there was a higher permeability at baseline while gluten was absent.

Eliminating all dietary gluten may be considered more of an aspirational goal than a fact, as this is difficult to attain even for highly motivated patients [52]. The GFD adherence rates are generally above 80–90% [53]. However, due to the wide use of wheat in most food ingredients, gluten can be difficult to avoid, resulting in accidental gluten exposure [54], as summarized in Table 1. These difficulties of the GFD were highlighted by the Determination of Gluten Grams Ingested and Excreted By Adults Eating Gluten-free (DOGGIEBAG) study. It involved 18 adults with biopsy-confirmed celiac disease who were on a GFD for 24 months and collected food (25% portions in a “doggie bag”), urine, and stool samples over 10 days. Although non-intentional gluten intake was reported, two-thirds had at least one sample that tested positive for immunogenic gluten peptides. This can be a reason that following a GFD, up to 30% of patients have persistent problems [46], and the therapeutic small-bowel villous atrophy is reported in 60% of patients, and the enteropathy may even persist [9,55].

A GFD has been reported to be more burdensome than treatments for type 1 diabetes, irritable bowel syndrome (IBS), and congestive heart failure [49]. A life-long strict GFD is a challenging task for the elderly, people with reading or language barriers, those with psychological impairment, and those with limited financial means. Dining out, social events, or using a school or professional canteen become real-life obstacles daily [56].

Surveys suggest that most CeD patients would be interested in medical therapy, not based on diet alone [57]. The goal of these future therapies will not be to act as adjuncts to a GFD for people with non-responsive CeD or refractory CeD but aim to allow those patients to consume gluten-without harm. Different interventions and pharmacological treatments for CeD have been developed in recent years. The comprehensive review completed by Segura et al. focuses on phase III and II clinical trials; it includes the developments in the degradation of gluten in the intestinal lumen, regulation of the immune response, modulation of intestinal permeability, and induction of immunological tolerance [58]. If there is interest in knowing about the non-dietary strategies for CeD, please refer to recent reviews [58,59]. Developing suitable, safe drugs for autoimmune disorders is complicated, and CeD is no exception. Since certain microorganisms can degrade gluten, creating nutritional supplements such as prolyl-endopeptidases (PEPs) seems to be an easy dietary intervention. Although, until now, there have been no such efficient products, it constitutes the mainstream strategy for nutritional interventions in CeD. Herein, we are interested in the emerging dietary approaches based on PEPs and extending them to modulating/shaping the patients’ microbiota with their potential benefit for the management and/or prevention of CeD. The selection of examples was based, in some cases, on historical relevance and, for others, was based on novelty to provide a generalist overview and promote new areas of research at the interface of the disciplines.

## 2. Dietary Interventions for Complementing the GFD and Beyond

### 2.1. Enzymes as a Nutritional Supplement Therapy for CeD

Several proteases and peptidases have been proven to degrade gluten in vitro and/or in vivo [60,61]. As aforementioned, mammalian gastrointestinal proteases partially digested immunogenic gluten sequences [33,62]. Therefore, the detoxification of gluten can theoretically be achieved by proteolytic fragmentation by oral enzymatic therapy. The idea is to inactivate gluten peptides in the human gastrointestinal tract by peptidase supplementation, thereby minimizing the amount of gluten peptides reaching the small intestine. The gluten-hydrolyzing enzymes produced by the *Rothia mucilaginosa* were have been identified as two structurally closely related *subtilisins* [63]. Previously, some of us reported the significant hydrolysis of wheat gliadin by Peptidase S9, isolated from the *B. tequilensis* strain [64]. Several gluten-detoxifying peptidases have been isolated from probiotic preparations involving *lactobacilli* [65,66], other microorganisms [63,67], and germinating cereals [68].

The withdrawal or modification of celiac peptides during food processing using enzymes is already commercialized. For example, a dietary supplement based on *Aspergillus niger*-Prolyl endopeptidase (PEP) can degrade gluten at a particular stage. However, it is not currently a treatment for CeD because it does not entirely break down gluten, and the resulting accumulation of gluten peptides in the duodenum has not been determined [69]. Another commercialized product is based on caricain, a proteolytic enzyme obtained from the papaya plant and papain. Previous studies have reported that caricain has the potential specificity to target gluten amino acid sequence and helps reduce gluten concentration during food processing [70,71]. However, to date, all the commercialized enzymatic cocktails are not prescribed for CeD patients.

Other prolyl endopeptidases (PEPs) isolated from *Myxococcus xanthus* and *Flavobacterium meningosepticum* showed the ability to hydrolyze toxic gliadin peptides significantly. However, the presence of immunopeptides has not been determined [72,73,74,75]. PEPs from *Sphingomonas capsulate,* showed complete hydrolysis of immunogenic gluten peptides after mixing with barley cysteine endoproteases [17,76]. Another interesting PEP is latiglutenase, in which experiments in subjects receiving 900 mg of latiglutenase led to improvements (*p*-values) in the severity of symptoms relative to placebo-dosed subjects for week 12. The reduction in symptoms trended higher for more symptomatic patients [77]. However, previous randomized phase 2 trials were conducted with latiglutenase (IMGX003, formerly ALV003) (ClinicalTrials.gov, NCT03585478), and they reported contradictory findings regarding its effect on villous atrophy and clinical symptoms, showing only 88% gluten hydrolysis efficiency [78,79]. Nowadays, a phase I clinical study is being conducted to evaluate the bacterial endopeptidase TAK-062 that simultaneously targets proline and glutamine peptide motifs in the stomach (ClinicalTrials.gov Identifier: NCT05353985). TAK-062 is a second generation of the engineered endopeptidase kuma030 [80]. When healthy individuals ingested TAK-062 before a complex meal containing 1–6 g gluten, it was observed that after 20–65 min post-TAK-062 ingestion, 97–99% of the gluten was degraded as a measure in aspirate samples from the stomach [81]. The calculated remaining gluten showed a median amount of up to 38 mg. To our knowledge, this is the first glutenase that showed this high gliadin hydrolysis efficiency in vivo. This has a potential clinical relevance since amounts as low as 10 mg of gluten may be able to trigger the immunological cascade [82,83]. Yet, these data showed the high efficiency of TAK-062, and further studies in CeD patients are in progress to test the efficiency of Tak-062 in degrading inadvertent gluten exposure (ClinicalTrials.gov Identifier: NCT05353985).

### 2.2. Human Microbiota and Dysbiosis in CeD

During the co-evolution of humans and microbes, thousands of bacterial species have colonized the human body. The vast amount of microbial presence in the host’s body is termed “normal flora,” “microbiota,” or “microflora” [84,85,86]. The microflora consists of bacteria accompanied by fungi, archaea, viruses, and protozoans [87,88,89]. This colonization occurs at birth, covering every human body surface, including the ear, oral cavity, respiratory tract, genitourinary tract, and gastrointestinal (GI) tract [86,90]. The GI tract is loaded with a plethora of molecules providing nutrition to microbes, facilitating heavy colonization of harmful and beneficial microbes.

The indigenous gut bacteria maintain themselves and protect the host against freshly ingested microbes, including pathogens. It is an essential immune mechanism in the host, referred to as the “barrier effect” or “colonization resistance” [91,92]. Indigenous microbes present in the gut microflora were also reported to regulate the development of the structure and morphology of the GI tract.

Each healthy individual has a unique gut microbiota [93]. The two major bacterial phyla are *Firmicute*s (*Bacillota*) and *Bacteroidetes (Bacteroidota)*, which are 90% of the whole gut microbiota [94]. The *Firmicutes* species is composed of ≥ 200 different genera, and *Clostridium* genera are 95% of the *Firmicutes* phyla. Bacteroidetes consist of predominant genera such as *Bacteroides* and *Prevotella*. *Actinobacteria, Proteobacteria, Fusobacteria*, and *Verrucomicrobia* are the next most numerous phyla, which are reported in a “healthy gut microbiota composition” [95].

Recently, many findings have reported that gluten metabolism is closely related to the GI microbiota [96,97,98]. The detailed mechanisms of microorganisms that play a protective role in CeD pathogenesis are broad. They comprise the metabolism of the triggering antigen (e.g., gliadin), increased intestinal barrier permeability, and inflection of innate and adaptive immune responses [99]. In 2016, Caminero et al. reported that the bacteria in the human GI tract could hydrolyze gluten in vivo and efficiently reduce its immunogenicity [100]. *Faecalibacterium prausnitzii*, *Roseburia intestinalis*, and *Eubacterium hallii* demonstrated a capability to restore and improve intestinal permeability [101]. Furthermore, orally administered bacteria, *Lactococcus lactis*, has been reported to induce antigen-specific tolerance in an experimental animal model [102]. Moreover, gluten hydrolyzing actions by dental plaque bacteria were reported [103], showing that the host’s indigenous bacteria could be able to degrade gluten.

Interestingly, microbial dysbiosis has been identified in patients with active CeD, which was exquisitely reviewed by Girvoban A. et al. in 2017 [104]. Their main conclusion was that both duodenal and colonic dysbiosis are associated with CeD. They reported that the most frequent Gram-negative bacterial species isolated from CeD patients were: *Bacteroides* spp., *Salmonella* spp., *Shighellaspp*, *Klebsiella* spp., *Neisseria* spp., and *Prevotella* spp. Although CeD is associated with a decrease in the number of Gram-positive bacteria, pathogenic Gram-positive species, such as *Clostridium* spp., *Staphylococcus* spp., and *Actinomyces* spp., were isolated from CeD patients. Of note is that bacterial virulence features are considered higher in CeD patients. Among them, it was reported that a peculiar *Neisseria flavescens* strain was identified in adults affected by CeD [105,106], using the 16S rRNA technique for duodenal and oropharyngeal samples from celiac patients and control subjects. This *Neisseria flavescens* strain, isolated from the CeD patients, induced an immune-inflammatory response in human and murine dendritic cells, both in CaCo-2 cells and in ex vivo duodenal mucosal explants of control subjects, thereby suggesting that it could play a role in CeD [105]. Leonard et al. reported that intestinal dysbiosis is associated with CeD onset in infants. They performed a prospective metagenomic analysis of the gut microbiota of infants at risk of CeD to track shifts in the microbiota before CeD development. The cross-sectional analysis at CeD onset identified an altered abundance of six microbial strains of *B. longum* and several metabolites between cases and controls but no change in microbial species or pathway abundance [83]. One of the main findings was the dysregulated interaction between the genus *Bifidobacteria* and butyrate-producing bacteria *Faecalibacteriumprausnitzii*, and *Clostridium clostridioforme* which could be critical in the development of CeD. Additionally, they reported new microbes (e.g., *Porphyromonas* sp.), pathways (e.g., high mannose–typeN-glycan biosynthesis), and metabolites (e.g., serine) that can be CeD-specific biomarkers. In another study, it was found that the stool microbiota of children with CeD active showed a significant abundance of *Bacteroides*-*Prevotella*, *Akkermansia*, and *Staphylococcaceae* compared with healthy controls. Interestingly, at the symptom level, the authors found a significantly increased mean relative abundance of *Bacillaceae* and *Enterobaeriaceae* in patients with abdominal pain. Meanwhile, those patients with diarrhea had a significantly reduced mean relative abundance, particularly of *Akkermansia.* The main conclusion was that CeD active patients’ microbiota differed from controls, where a pro-inflammatory microflora was found. Following the microbiota of such patients in GFD could shed light on the role of gluten in the observed disbalance [107].

In this direction, a recent report from Palmieri et al. described that adherence to GFD restored the alpha biodiversity, a measure applicable to a single sample of the gut microbiota in celiac people, showing a non-dysbiotic microbial composition. However, the microbial composition at beta diversity, a measure of the similarity or dissimilarity of two communities, resulted in differences from healthy controls. In concrete, the authors found that the microbial composition of the CeD subjects in GFD was decreased in several taxa, namely *Bifidobacterium longum* and several belonging to the *Lachnospiraceae* family. In contrast, the *Bacteroides genus* was found to be more abundant. Predicted metabolic pathways among the CeD bacterial communities revealed an important role in tetrapyrrole biosynthesis [108].

### 2.3. Probiotics

Probiotics are live microorganisms that have demonstrated beneficial effects on human health after being administered in adequate amounts by restoring the composition of the gut microbiome to prevent gut microbiota dysbiosis and improve immunity [109,110,111,112]. In this regard, probiotic bacteria are constantly being studied, and their applications are also being considered in promising adjuvant treatments for various intestinal diseases, including CeD [113,114]. Most of the probiotic bacteria belong to the genus *Lactobacillus* and *Bifidobacterium*. They are considered “Generally Recognized As Safe’’ (GRAS) by the United States Food and Drug Administration (USFDA) [115]. However, some researchers reported that several *Bacillus* sp. also fulfill the essential probiotic characteristics, such as resistance to antibiotics as well as acid, bile salt, and sodium chloride tolerance, and produce a group of antimicrobial peptides with a broader inhibition spectrum [116]. Probiotic *Lactobacillus* sp. and *Bacillus* sp. isolated from different sources are mainly used as probiotic candidates because they are generally safe and cost-effective [117]. Both of these species are usually found in abundance in the upper GI tracts of both humans and animals. De Angelis et al. reported the formulation of commercial enzymes with microbial consortia of *Lactobacillus* and *Bacillus*, named consortia I: *Lactobacillus (Lp.) plantarum*, *(Lc.) paracasei*, *Bacillus subtilis*, *Bacillus pumilus*, and consortia II: *Lp. plantarum*, *Lc. Paracasei*, *Limosilactobacillusreuteri*, *Bacillus megaterium*, *B. pumilus*, showed hydrolysis of gluten to non-immunogenic and non-toxic peptides under GI conditions. These findings state that both microbial consortia can detoxify immunogenic gluten peptides and may be used to improve the intestinal digestion of CeD and gluten-sensitive patients [113].

A curative measure of probiotics can help by preventing and treating conditions like IBD (e.g., Crohn’s disease and ulcerative colitis), autoimmune diseases (e.g., rheumatoid arthritis), CeD and lactose intolerance, IBS, vaginal infections (e.g., candida or thrush), and atopic dermatitis [118]. Probiotic consumption also helps to reduce diarrhea and allergies. Probiotics found in dairy and meats reduced low-density lipoprotein (LDL) levels, killed the bacteria that caused tooth decay, and lessened the harmful effects of gingivitis. Probiotics also stimulate, modulate, and regulate the host’s immune response, gastrointestinal hormone release, and brain-behavior through bidirectional neuronal signaling [119,120]. Probiotics have physiological functions that improve the host environment’s health, regulate microbes, and are also supportive in combating obesity and being overweight [121]. There are some examples where probiotic prophylaxis was given to patients with severe acute pancreatitis and the probiotics caused significantly more severe side effects [122]. Thus, the exact mechanisms of the health-promoting effects of probiotics remain elusive. However, it would be of great significance to explore membrane and extracellular proteins/enzymes and other biomolecules of probiotics [123]. These bacteria produce diverse compounds such as organic acids, enzymes, bacteriocins, antimicrobial compounds, exopolysaccharides, secreted low-calorie sweetening molecules, and nutraceuticals [124]. Probiotics are now a rising field for food manufacturers with remarkable growth potential. As it involves the ingestion of live probiotic bacterial cultures, it enhances the intestinal microflora. The importance and success of probiotics in the overall market will depend on the effectiveness of the probiotic strain or cultures used. The food products which contain probiotics and prebiotics affect the functionality of the foods, which results in the enhancement of the microflora that promotes gut health [125].

Recently, many researchers have focused on screening gliadin-cleaving proteolytic activity from probiotic strains. *Lactobacilli* and *Bifidobacterium* are considered essential intestinal microbiota having beneficial effects on human health and are widely used in the formulation of probiotic products. Therapy with probiotics containing bacteria that can degrade gluten could be a possible new strategy for the complementary treatment of CeD patients [126]. *Bifidobacterium* species showed significant digestion of gluten protein and reduced cytotoxicity and pro-inflammatory responses [127]. A probiotic preparation (two strains of *Lactobacilli* and three strains of *Bifidobacteria*) hydrolyzes the gliadin peptides and protects intestinal cells from the toxic pro-inflammatory peptides [128]. Heeney et al. reported that low levels of probiotic *Lactobacilli* gave inconsistent findings in the microbiomes of adults and children showing active CeD [129]. However, many reports showing probiotic-induced beneficial effects using animal models with some features of CeD showed that probiotics could positively influence disease pathology through various mechanisms, as shown in Table 2.

Akobeng et al. reported that microbial imbalances that occur because of a GFD have led to the consideration of other therapeutic approaches, such as introducing probiotics to the GFD [134]. Probiotics play an important role in the restoration and modulation of gut microbiota. Indeed, gut microbiota composition influences the spectrum of gastrointestinal symptoms of CeD [107]. Marasco et al. reported that the use of probiotics in CeD could modulate the composition and functions of the microbiota, which may delay the CeD or prevent it. In the same study, they reported that after the administration of probiotic strains from *Bifidobacterium* and *Lactobacilli*, they restore gut microbiota and pre-digest gluten peptides in the intestinal lumen, reducing the inflammation associated with gluten intake, intestinal permeability, and cytokine and antibody production. These findings give ideas about improving symptoms and quality of life in patients treated with probiotics and GFD [135].

### 2.4. Prebiotics

Prebiotics are defined as a substrate that is selectively utilized by host microorganisms to confer a health benefit, for example, by stimulating one or more groups of gut-friendly microbes, mainly *Bifidobacterium* and *Lactobacillus*. Examples are substances in foods such as garlic, onions, artichokes, and others. Eating adequate amounts of these dietary foods might be necessary to have the beneficial “bifidogenic” effect. Another alternative is to take a prebiotic supplement to achieve the most favorable levels. In addition, prebiotics are resistant to hydrolysis by digestive enzymes and are not absorbed in the upper part of the gastrointestinal tract, reaching the large intestine where they stimulate certain microorganisms’ growth [136]. Different compounds have been tested to determine their function as prebiotics. Fructo-oligosaccharides (FOS), galacto-oligosaccharides (GOS), and trans-galacto-oligosaccharides (TOS) are the most common examples of prebiotics. The fermentation of prebiotics by gut microbiota produces short-chain fatty acids (SCFAs), including lactic acid, butyric acid, and propionic acid [137].

Drabinska et al. reported that a prebiotic, namely oligofructose-enriched inulin, significantly increased the *Bifidobacterium* count in the gut of celiac children with no adverse effects. This investigation focuses on a possible causative role of gut dysbiosis in CeD, although the exact mechanism remains unclear [138]. Studies are also focused on using a low fermentable oligo-, di-, monosaccharides, and polyols (FODMAP) diet, which are low short-chain polysaccharides like fructans, lactose, mannitol, sorbitol, etc. These sugars are difficult to digest, resulting in fermentation in the bowel and flatulence, and are implicated in causing some of the symptoms of IBS [139]. The low FODMAP diet may benefit CeD, especially those with functional IBS-like symptoms [140,141], but the molecular mechanism remains elusive.

Chen et al. reported a probable mechanism of action of prebiotics on the intestinal epithelial cell line, Caco-2, using a prebiotic blend composed of FOS, GOS, inulin, and anthocyanins in co-incubation with *Salmonella typhimurium*. In addition, the authors studied post-infectious IBS models in C57BL/6 mice. The study showed that the prebiotic blend significantly decreased pro-inflammatory cytokine production in both infected Caco-2 cells and post-infectious IBS models [142]. An excellent review from Marasco et al., taking into account randomized controlled trials, cross-sectional studies, and eminent reviews on the topic, shows that the inclusion of inulin as prebiotics in GFD can stimulate the growth of potentially healthy bacterial strains such as *Bifidobacterium* and *Lactobacillus* [135]. These authors also mentioned that most studies on CeD patients were performed with inulin; therefore, investigating prebiotics in CeD could be a fascinating area of study.

### 2.5. Synbiotics

Synbiotics are a combination of probiotics and prebiotics. These synbiotics contain probiotics, which are beneficial bacteria, and prebiotics, which are indigestible products for improving the growth of beneficial bacteria. In the sense that a product in which a prebiotic is specifically added favors the wanted probiotic’s growth. For example, fermented dairy products such as yogurt are synbiotic food products. The most common synbiotics include FOS and *Bifidobacteria;* inulins and *Lactobacillus*; and *Bifidobacteria*, and *Lactobacilli* with FOS [143,144]. Wilms et al. reported that synbiotic dietary strategies might be used to improve intestinal barrier functions. They reported that when 20 healthy adult individuals were supplemented with synbiotic supplementation Ecologic^®^ 82S + 10 g Fructo-oligosachharides P6(FOS P6) every day for two weeks, the individuals reported increased stool frequency. The intestinal permeability under basal and indomethacin-induced stressed conditions was determined, showing that these synbiotics neither affect the intestinal permeability, immune function, or gastrointestinal symptoms under basal or indomethacin-induced conditions [145].

Demiroren et al. reported that when children with potential CeD were supplemented with synbiotics containing a multi-strain of *Lactobacillus* and *Bifidobacterium* for 20 days, they showed a decrease in anti-tTG levels as compared with the control group without the supplementation with the synbiotics [146].

Ugural and Akyol questioned the role of pseudocereals, i.e., amaranth, quinoa, and buckwheat, in symbiotic formulations for treating dysbiosis in general and inflammation-mediated chronic disorders such as CeD [147]. They critically reviewed recent investigations in this relatively new area. They reported that in using cultures or naturally fermented pseudocereals, good substrate properties for probiotic bacteria were observed, increasing the amount of *Peptoclostridium*, *Prevotellaceae*, *Lactobacillus*, *Bifidobacterium*, *Enterococcus*, and *Eubacteriaceae*, and the increase in the synthesis of short-chain fatty acids due to their prebiotic effects. They mentioned that studies showing the prebiotic effects of amaranth, the pseudocereal with the highest protein content (13.56 g/100 g), are limited. One interesting experiment in this direction has been reported by Gullon et al. In this study, in vitro digested amaranth and quinoa grains were inoculated into fecal samples collected from healthy individuals. After incubation, microbiota varieties such as *Bifidobacterium* spp., *Lactobacillus–Enterococcus*, *Atopobium*, *Bacteroides–Prevotella*, *Clostridium coccoides–Eubacterium rectale*, *Faecalibacterium prausnitzii*, and *Roseburia intestinalis* were detected. In addition to this bacterial diversity, short-chain fatty acids such as acetate, propionate, and butyrate also increased. At the end of the study, it was demonstrated that quinoa and amaranth might have prebiotic activity [148].

### 2.6. Postbiotics

Postbiotics are products secreted by living bacteria or released after their lysis, for instance, molecules such as SCFAs, lactic acid, and bioactive peptides, among other metabolites. It can also be extended to protein compounds, hydrogen peroxide (H_2_O_2_), bacteriocins, organic acids, exopolysaccharides, and enzymes [149,150]. When postbiotics are administered in adequate amounts, they help improve the host’s health. Nevertheless, to date, the exact mechanisms of improvement have not yet been completely unfolded. The advantage of using postbiotics rather than probiotics concerns higher stability and safety: postbiotics do not contain any living bacteria and hence harbor no risk of microbial infection and translocation [151].

Postbiotics have been used in in vitro experiments in Caco-2 cells to analyze their ability to prevent gliadin and gliadin peptides’ effects on Caco-2 cells. Sarno et al. reported that postbiotics CBA L74, supernatant from *Lactobacillus paracasei*, could reduce gliadin peptides’ entrance into Caco-2 cells [152]. In this direction, recently, Conte et al. investigated the beneficial postbiotic effect from *Lactobacillus paracasei* CBA L74, both in Caco-2 cells and in vitro on CeD organoids after stimulation with pepsin-trypsin gliadin (PTG) digest or the cytotoxic 13-mer gliadin peptide. The postbiotic prevented the gliadin-induced activation of the inflammatory response as measured by activation markers NfkB and ERK phosphorylation and activation of mTOR signaling, and it was capable of inhibiting the gliadin-induced reduction of the autophagy pathway. Hence, *Lactobacillus paracasei* CBA L74 postbiotics decreased the gliadin-induced inflammatory response and stimulated autophagy, which has an important role in intestinal homeostasis [153]. Another selected report from Freire et al. used an in vitro model of organoids from non-celiac individuals and celiac patients to study the pathogenesis of CeD. In this study, they also investigated the effects of three postbiotics, butyrate, lactate, and polysaccharide A from *B. fragilis*. They found that these molecules could modulate the intestinal responses to gluten. The authors showed an increase in paracellular permeability that was already present at baseline in CeD organoids. In particular, butyrate and polysaccharide A could restore CeD barrier function through increased expression of the tight junction sealing molecule claudin-18. Likewise, incubation of the CeD organoids with gliadin induced immune activation (expression of IL-15 and IFN gamma) that was decreased by butyrate and lactate [51].

### 2.7. Viruses

Numerous publications exist on the human microbiome and the place of the corresponding dysbiota in specific human chronic conditions. The community of viruses in the gastrointestinal tract is named virome, and its role in health and disease is a fascinating new area of research [154].

The knowledge about the ecology of gut viruses is limited yet. Still, gut viruses outnumber microbes in a ratio of 10:1 [155]. The microbiome cannot maintain a homeostatic equilibrium without the gut phageome (a collection of bacteriophages).

The gastrointestinal virome biodiversity changes along with the human life cycle. With aging, the phage load decreases, while the abundance and complexity of the microbial populations increase substantially. It seems that intestinal bacterial composition and diversification occur at the expense of the virome communities [156]. In humans, viral dysbiosis in IBD has been reported, so it is not a surprise that children with CeD, which is also an inflammatory enteropathy, show a statistically significant viral dysbiosis by metagenomic analysis. In this sense, it was found recently that viral dysbiosis in children newly diagnosed with CeD before starting the GFD [157]. It was already reported that the lower initial diversity of the human gut virome leads to a more pronounced effect of the GFD on its composition [158], showing the impact of the GFD on the dynamics of the gut virome.

Some phages have been proposed as new prebiotics and are undergoing clinical trials to prove safety, tolerability, and efficacy. In a short intervention of 28 days, phages did not globally disrupt the microbiota. However, in response to the intervention, specific populations were altered as the members of the butyrate-producing genera increased. The authors concluded that bacteriophages could selectively reduce target organisms without causing global gut microbiome disruption [159].

In this direction, it has been hypothesized that phage therapy may represent a new strategy for treating CeD. Their role could be to select microbes that digest gluten or lack glutenase capacity, thus modifying the luminal gluten load or modifying the transglutaminase activity. Lerner et al. have presented different potential interventions [160]. Studies with functional analyses to define the relationship of bacteriophages to bacteria and to clarify the role of viruses in CeD might lead to the development of additional treatment options. A funded proof of concept project focusing on altering human gut microbes to treat gluten-related disorders is now advancing in this direction. The project’s objectives include engineering *Bifidobacterium*-targeting templated bacteriophages capable of infecting *B. longum* to express a gluten-degrading enzyme from *Sphingomonas capsulata* and the introduction of the glutenase-expressing phage into a *B. longum* in an in-vitro biofilm model [161]. The technology readiness levels (TRLs) is a validated method of 9 stages to estimate the maturity of technologies [162]. The use of viruses for therapeutic interventions in gastroenterology is currently in stage 1, when the basic principles are observed. Any actual clinical application of viruses in CeD therapy is still quite far away, but it is worth investigating what is going on and monitoring the advent of potential “viromebiotics”.

## 3. Perspectives

Many findings have proven a close relationship between probiotics, prebiotics, synbiotics, and postbiotics with intestinal flora and immunity concerning CeD, and “viromebiotics” is a plausible new area of research to complete the whole “biotics” scenario. Instead of other non-dietary strategies, these “Pro-Pre-Syn-Post” biotics might be an appropriate and bio-safe complementary dietary therapy against CeD. The complete molecular mechanisms of these “biotics” actions are in their infancy and require more basic research. We envisaged that the future metabolomic approach would provide insight into the knowledge of the molecular mechanisms of these “biotics” for a possible nutritional intervention for CeD in combination with the GFD or beyond it.

## Figures and Tables

**Table 1:** A Summary of the challenges of the gluten-free diet from [52,54,56].

**Gluten avoidance** Imprecise dietetic information More costly Poor palatability Risks when eating out of home
**Balanced diet** Insufficient fibers The flexibility of the diet Avoidance of disordered eating Weight control
**Social restrictions** Anxiety and social isolation cultural pressures Impaired quality of life needless limitations in daily life

**Table 2:** Selected examples of animal models.

Probiotic Cultures	Animal Models	Mode of Sensitization	Major Findings	References
*Lactobacillus casei*	Transgenic mice expressing the human DQ8 heterodimer	Chymotryptic digest of gliadin and cholera toxin	Enhanced the gliadin-specific response mediated by CD4 T cells.	[130]
*Lactobacillus casei*	Transgenic mice expressing the HLA-DQ8 molecule without endogenous mouse class II genes, non-transgenic for human CD4.	Wheat gliadin	*L. casei* can be effective in rescuing the normal mucosal architecture.	[131]
*Bifidobacterium longum* CECT 7347	Female weanling Wistar rats	Gliadin	Ameliorate the inflammation caused by gliadin.	[132]
*Bifidobacterium longum* CECT 7347	Female, weaning Wistar rats	IFN-g and fed gliadin	*Bifidobacterium longum* attenuates the production of inflammatory cytokines, and the CD4 C T-cell mediated immune response.	[133]
*Saccharomyces* *boulardii* KK1strain	BALB/c mice	Gluten-containing commercial food pellets	Improved enteropathy development In association with a decrease of epithelial cell CD71 expression and local cytokine production.	[15]
